# The impact of coronavirus outbreak on breastfeeding guidelines among Brazilian hospitals and maternity services: a cross-sectional study

**DOI:** 10.1186/s13006-021-00377-1

**Published:** 2021-03-31

**Authors:** Walusa Assad Gonçalves-Ferri, Fábia Martins Pereira-Cellini, Kelly Coca, Davi Casale Aragon, Paulo Nader, João Cesar Lyra, Maryneia Silva do Vale, Sérgio Marba, Katiaci Araujo, Laura Afonso Dias, Daniela Marques de Lima Mota Ferreira, Gislayne Nieto, Lêni Marcia Anchieta, Rita de Cássia Silveira, Marta David Rocha de Moura, Valdenise Martins L. Tuma Calil, Viviane Christina Cortez Moraes, João Henrique Carvalho Leme de Almeida, Maurício Magalhães, Thaise Cristina Branchee Sonini, Juliane Barleta Javorsky, Érica Lobato Acaui Ribeiro, Rodrigo Ferreira, Louise Dantas Cavalcante de Almeida, Rosângela Garbers, Gabriella Maset da Silva Faria, Anelise Roosch, Ana Ruth Antunes de Mesquita, Rebecca Meirelles de Oliveira Pinto, BRACOVID Project Collaborative Group

**Affiliations:** 1 Department of Pediatrics, Ribeirão Preto Medical School– University of São Paulo, Ribeirão Preto, SP Brazil; 2 Department of Pediatrics, Universidade Federal de São Paulo, São Paulo, SP Brazil; 3 Department of Pediatrics, Curso de Medicina da ULBRA, Universidade Luterana do Brasil, Porto Alegre, RS Brazil; 4 Department of Pediatrics, Neonatology Discipline, Faculdade de Medicina do Campus de Botucatu, UNESP, Botucatu, SP Brazil; 5 Department of Pediatrics, Universidade Federal do Maranhão, São Luís, MA Brazil; 6 Department of Pediatrics, Universidade Estadual de Campinas- UNICAMP, Campinas, SP Brazil; 7 Department of Pediatrics, Hospital Aliança, Salvador, BA Brazil; 8 Department of Pediatrics, Maternidade Lilia Neves, CEPLIN - Instituto de pediatria e neonatologia ltda, Campus dos Goitazes, RJ Brazil; 9 Departments of Pediatrics, Federal University of Uberlandia, Uberlândia, MG Brazil; 10 Department of Pediatrics, Hospital e Maternidade Santa Brígida, Curitiba, PR Brazil; 11 Department of Pediatrics, Universidade Federal de Minas Gerais, Belo Horizonte, MG Brazil; 12 Department of Pediatrics, Universidade Federal do Rio Grande do Sul, Porto Alegre, RGS Brazil; 13 Department of Pediatrics, Hospital Materno Infantil de Brasília, Brasília, DF Brazil; 14 Department of Pediatrics, Instituto da Criança do Hospital das Clínicas da FMUSP, São Paulo, SP Brazil; 15 Department of Pediatrics, Maternidade Perinatal Laranjeiras, Rio de Janeiro, RJ Brazil; 16 Department of Pediatrics, Instituto Nacional de Saúde da Mulher, da Criança e do Adolescente Fernandes Figueira – Fundação Oswaldo Cruz, Rio de Janeiro, RJ Brazil; 17 Department of Pediatrics, Hospital Central da Santa Casa de Misericórdia de São Paulo, São Paulo, SP Brazil; 18 Department of Pediatrics, Hospital Maternidade Carmela Dutra, Florianópolis, SC Brazil; 19 Department of Pediatrics, Complexo Hospital de Clínicas UFPR, Curitiba, PR Brazil; 20 Department of Pediatrics, Hospital da Clínicas da Faculdade de Medicina de Marília -Unidade Materno Infantil, Marília, SP Brazil; 21 Department of Pediatrics, Universidade Do Estado Do Amazonas, Platô do Piquiá, Boca do Acre, AM Brazil; 22 Department of Pediatrics, Maternidade Climério de Oliveira- UFBA, Salvador, BA Brazil; 23 Department of Pediatrics, Maternidade Nossa Senhora de Fátima, Curitiba, PR Brazil; 24 Department of Pediatrics, Faculdade de Medicina de São José do Rio Preto-FAMERP, São José do Rio Preto, SP Brazil; 25 Department of Pediatrics, Ribeirão Preto Medical School, Ribeirão Preto, São Paulo Brazil

**Keywords:** Breastfeeding, COVID-19, Public health, Skin-to-skin, Brazil, Breastfeeding guidelines, Breastfeeding practices, Milk bank

## Abstract

**Background:**

The World Health Organization recognizes exclusive breastfeeding a safe source of nutrition available for children in most humanitarian emergencies, as in the current pandemic caused by the Severe Acute Respiratory Syndrome Coronavirus 2 (SARS-CoV-2). Despite the Brazilian national guideline protecting breastfeeding practices, there are many concerns about protecting infants from their infected mothers. This study aimed to analyze how the Brazilian hospitals and maternity services promote and support mothers suspected or diagnosed with coronavirus disease (COVID-19).

**Methods:**

This is a descriptive cross-sectional and multicenter study which collected data from 24 Brazilian hospitals and maternity services between March and July 2020. Representatives of the institutions completed a questionnaire based on acts to promote and support breastfeeding, the Baby-Friendly Hospital Initiative, and Brazil’s federal law recommendations.

**Results:**

The results showed that in delivery rooms, 98.5% of the services prohibited immediate and uninterrupted skin-to-skin contact between mothers and their infants and did not support mothers to initiate breastfeeding in the first hour. On the postnatal ward, 98.5% of the services allowed breastfeeding while implementing respiratory hygiene practices to prevent transmission of COVID-19. Companions for mothers were forbidden in 83.3% of the hospitals. Hospital discharge was mostly between 24 and 28 h (79.1%); discharge guidelines were not individualized. Additionally, a lack of support was noticed from the home environment’s health community network (83.3%). Hospital and home breast pumping were allowed (87.5%), but breast milk donation was not accepted (95.8%). There was a lack of guidance regarding the use of infant comforting strategies. Guidelines specific for vulnerable populations were not covered in the material evaluated.

**Conclusions:**

In Brazil, hospitals have not followed recommendations to protect, promote, and support breastfeeding during the COVID-19 outbreak. The disagreement between international guidelines has been a major issue. The absence of recommendations on breastfeeding support during the pandemic led to difficulties in developing standards among hospitals in different regions of Brazil and other countries worldwide. The scientific community needs to discuss how to improve maternal and infant care services to protect breastfeeding in the current pandemic.

## Background

The coronavirus disease (COVID-19) caused by Severe Acute Respiratory Syndrome Coronavirus 2 (SARS-CoV-2) is a major public health concern and has led to thousands of deaths worldwide. On July 29, 2020, Brazil registered 2,483,191 infected people by SARS-CoV-2 and a mortality rate of 42.1 per 100,000 inhabitants [1, 2].

Despite recommendations for breastfeeding and other protective practices to minimize viral transmission, including temporary separation of mother and child during active infection, the precise numbers of maternal-infant transmission are not known due to the potentially high number of asymptomatic infected women who are not tested for COVID-19. Nevertheless, COVID-19 fatality among neonates and the infection during breastfeeding seem infrequent [3–5]. Although it cannot be excluded that an infant exposed to SARS-CoV-2 may develop COVID-19 and respiratory failure, the reported neonatal cases to date are generally mild with favorable outcomes [5, 6].

Breastfeeding improves both the mother’s and infant’s health and has a positive social and economic impact [7–10]. Based on current scientific knowledge, there is no evidence that breast milk from a mother with COVID-19 is a transmission vehicle [4, 11–14]. The guidelines for the management of the mother-infant dyad exposed to SARS-CoV-2 recommend a complex hospital organization and infection control measures for neonates during breastfeeding; however, there are few references on promoting and supporting this practice in the pandemic [15–27].

Since breastfeeding has an essential impact on the country’s socio-economic organization, Brazil has robust policies to promote this practice. The measures to promote and support breastfeeding recommended by Brazilian public health policies are encouraged and implemented in maternal and infant care services [25–27].

However, when we consider the mother-infant dyad suspected or diagnosed with COVID-19, there was a diversity in recommendations early in the pandemic and a lack of information on how Brazilian hospitals could prepare themselves to maintain the practices of supporting breastfeeding amid the pandemic. This study aimed to analyze how Brazilian hospitals and maternity services promote and support breastfeeding to mothers suspected or diagnosed with COVID-19.

## Methods

### Design

A descriptive, cross-sectional, and multicenter study was conducted from March to July 2020.

### Participants

Thirty university hospitals or centers related to research, education, and extension in pediatric care were offered participation from different regions of the country, represented by the local coordinate services. All maternity hospitals in each Brazilian region which offered care for COVID-19 patients were invited to participate. Hospitals that did not respond or refused to participate were excluded from the study.

### Questionnaire development, data collection and analysis

We prepared a structured questionnaire based on the guidelines from the Baby-Friendly Hospital Initiative (BFHI). In addition, we included information about the Federal Law No. 11,108, of April 7, 2005, Companion Law, which states that Brazilian National Health Systems (SUS), whether owned or affiliated, are obliged to allow pregnant women the right to be accompanied throughout the labor, delivery, and postpartum period. The companion may be the baby’s father, the current partner, the mother, a friend, or another person of her choice.

We sent a structured questionnaire to the hospitals’ coordinators electronically, between 1 March and 25 July 2020. An affirmative response indicated that the guideline recommended a particular variable and a negative response indicated that the guideline recommended against a particular variable (RA/contraindicated) or that there was no hospital guideline regarding the variable (NR) for suspected/positive COVID-19 dyads.

### Measures

We investigated the degree to which the institutions recommended breastfeeding support measures in their own or individual institutional guidelines.

The recommendations in the delivery room, rooming-in practice, and home environment were evaluated. The frequencies were recorded as whether the breastfeeding support recommendations were in the guidelines (yes) or whether the recommendations were not in the guidelines (no), in accordance with the local guidelines for the management and breastfeeding promotion of the mother-infant dyad exposed to SARS-CoV-2, based on national public health programs, specially the BFHI [15–27].

#### Delivery room

The variables evaluated regarding breastfeeding promotion were skin-to-skin contact, breastfeeding in the first hour, presence of companions, personal protective equipment (PPE) for the patient, neonatal baths, and other recommendations to avoid the SARS-CoV-2 infection.

#### Rooming-in

The variables evaluated were the following: shared decision-making with the family about breastfeeding, responsive feeding, availability of an individual room, dyad separation, presence of a companion, hygiene and distancing measures, psychological support availability, hospital orientation offered, and hospital discharge in 24 to 48 h.

The hygiene measures included mother’s usage of a surgical mask, hand washing before breastfeeding, mother’s washing their face before breastfeeding, breast cleansing before breastfeeding, and changing clothes or wearing an apron when breastfeeding. Regarding distancing guidelines, we considered the recommendation that the infant should be placed in the crib at least 2 m from the mother during periods of non-breastfeeding.

#### Home environment recommendations

The variables evaluated were the following: recommendations on promoting breastfeeding, responsive feeding, comforting techniques, neonatal care delegation, mixed feeding (breast milk and infant formula), hygiene and distancing measures, guidance material provision, home visit, telemedicine use, primary reference health service support, newborn screening in the hospital, and psychological call center availability.

Hygiene measurements were divided into hygiene before and after breastfeeding. Regarding distancing recommendations, dyad separation, dyad distancing during periods of non-breastfeeding, and distancing between siblings were evaluated and using a separate room.

#### Breast pumping and human milk donation recommendations

The recommendations evaluated were related to hospital pumping, bedside pumping, home pumping, usage of mother’s fresh breastmilk, pasteurization of breast milk, use of a feeding line at the breast, cup feeding, and permission to donate milk. The feeding line technique was characterized by the infant feeding with milk expressed by the mother, administered by a thin tube attached to the nipple, during breastfeeding.

#### National and international guidelines

All variables observed were related to the Brazilian societies and some international guideline recommendations available in the literature: the Brazilian Society of Pediatrics (SBP), Brazilian Network of Human Milk Banks (rBLH), World Health Organization (WHO), Center for Disease Control and Prevention (CDC), United Nations Children’s Fund (UNICEF), Royal College of Obstetricians and Gynaecologists (RCOG), Society of Italian Neonatology (SIN) endorsed by Union of European Neonatal and Perinatal Societies (UENPS) and Academy of Breastfeeding Medicine (ABM) [15–27]. The international guidelines were consulted in July 2019. We used the international recommendations existing in this period because it was when Brazilian hospitals developed their recommendations.

Data were analyzed using descriptive statistics.

## Results

Twenty-four hospitals, distributed across four Brazilian regions, participated by answering the questionnaire: 13 hospitals from the Southeast, six from the South, one from the Midwest, one from the North and three from Northeast. All the hospitals performed maternal screening through real-time reverse transcription polymerase chain reaction (RT-PCR) on mothers with symptoms of COVID, and 18 (75%) performed the newborn screening RT-PCR before discharge.

Among the 24 hospitals, 12 (50%) were BFHI certificated by the Ministry of Health, and only two (8.3%) did not have a local guideline for the management of the mother-infant dyad exposed to SARS-CoV-2. Twenty of the services evaluated are public university hospitals. The practices adopted in delivery rooms are described in Table 1.

**Table 1:** Recommendations in delivery room for the management of the mother-infant dyad exposed to SARS-CoV-2

Recommendation	Brazilian hospital guidelines existence (*n* = 24)		Available recommendations in Brazilian and international literature [15–27]
	Yes (%)	No (RA/NR -%)	
Skin-to-skin contact	5 (20.3)	19 (79.1)/RA	RCOG, WHO, UNICEF allow; SBP does not recommend; ABM and CDC suggest separation.
Breastfeeding in the delivery room (first hour)	3 (12.5)	21 (87.5)/RA	ABM, CDC, SBP, UENPS do not specify; RCOG considers mother’s choice; WHO and UNICEF allow.
Companion in the delivery room	11 (45.8)	13 (54.1)/RA	SBP recommends healthy adult, RCOG recommends for mother’s mental health; CDC suggests virtual companion; ABM, CDC, UNICEF, WHO, UENPS and SBP do not specify.
PPE to mother	5 (20.3)	19 (79.1)/NR	All guidelines recommend.
Neonatal bath	11 (45.8)	13 (54.1)/NR	SBP recommends an individualized bath routine; RCOG, ABM, CDC, WHO, UNICEF and UENPS do not specify.

*RA* Recommended against, *NR* There was no description of the recommendation in guidelines, *ABM* Academy of Breastfeeding Medicine, *rBLH* Brazilian Network of Human Milk Banks, *SBP* Brazilian Society of Pediatrics *CDC* Centers for Disease Control and Prevention, *PPE* Personal Protective Equipment, *RCOG* Royal College of Obstetricians and Gynaecologists, *UENPS* Union of European Neonatal and Perinatal Societies, *UNICEF* United Nations Children’s Fund, *WHO* World Health Organization

Most of the hospitals did not recommend the actions to promote breastfeeding in the delivery room, did not perform skin-to-skin contact (79.1%), and did not encourage breastfeeding in the first hour after birth (87.5%).

Distancing and breastfeeding recommendations were carried out in all hospitals. Only one hospital (4.1%) recommended dyad separation. The decision to breastfeed was shared with the mother in 75% of the evaluated hospitals, however a companion was forbidden in the majority of the centers (83.3%; Table 2).

**Table 2:** Recommendations for rooming-in for the management of the mother-infant dyad exposed to SARS-CoV-2

Recommendation	Brazilian hospital guidelines existence (*n* = 24)		Available recommendations in Brazilian and international literature [15–27]
	Yes (%)	No (RA/NR-%)	
Shared decision-making	18 (75.0)	6 (25.0)/NR	RCOG, CDC and ABM encourage shared decision-making; WHO suggests the dialog but reinforces the need of speaking about the benefits on breastfeeding; UNICEF, UENPS and SBP do not specify.
Feeding on demand	23 (95.8)	1 (4.1)/NR	None of the guidelines specifies.
Individual room for dyad	15 (62.5)	9 (37.5)/NR	rBLH and UENPS recommend individual room; RCOG and ABM offers as an option; WHO and UNICEF do not specify; CDC recommends separation.
Dyad separation	1 (4.1)	23 (95.8)/RA	UNICEF, RCOG and WHO do not recommend; CDC recommends; UENPS advises against separation, allows only if mother is symptomatic or the hospital logistics forbids it; SBP and ABM do not specify.
Companion room	4 (16.6)	20 (83.3)/RA	CDC limits visits and suggests rules; UENPS and rBLH suspend visits; SBP, RGCO and ABM suggest healthy adult to help; WHO and UNICEF do not specify.
Use of mask	23 (95.8)	1 (4.1)/NR	RCOG, ABM, UNICEF, CDC, UENPS and SBP recommend.
Washing hands	23 (95.8)	1 (4.1)/NR	RCOG, ABM, SBP, WHO, UNICEF, CDC and UENPS recommend.
Washing the face	4 (16.6)	20 (83.3)/NR	WHO, CDC, RCOG, SBP, ABM, UNICEF and UENPS do not specify.
Cleaning the breast	7 (29.1)	17 (70.8)/NR	WHO, CDC, RCOG, SBP, ABM and UENPS do not specify; UNICEF recommends if mother is coughing.
Changing the clothes	2 (8.3)	22 (91.6)/NR	WHO, UNICEF, CDC, RCOG, SBP, ABM and UENPS do not specify.
2 m distance	24 (100)	0 (0)/RA	ABM, CDC, UENPS and rBLH recommend distance; WHO suggests precaution, however keeping mothers and infants together day and night. RCOG, UNICEF does not specify.
Psychological support	23 (95.8)	1 (4.1)/NR	ABM, SBP, UNICEF, WHO and UENPS do not specify; CDC and RCOG recommend.
Home environment recommendations for the mother	24 (100)	0 (0)/NR	ABM and SBP do not specify; rBLH recommends if mother is insecure; WHO, RCOG, CDC, UNICEF and UENPS recommend.
Home environment recommendations for the family	18 (75)	6 (25)/NR	ABM, WHO, UNICEF, UENPS and SBP do not specify; RCOG and CDC recommend.
Hospital discharge (24-48 h)	19 (79.1)	5 (20.8)/RA	UENPS recommends discharge in 48 h, if hospital is crowded; if it is not, it recommends 1 week in the hospital to repeat the swab test; WHO, CDC, RCOG ABM, SBP and UNICEF do not specify.

*RA* Recommended against, *NR* There was no description of the recommendation in guidelines, *ABM* Academy of Breastfeeding Medicine, *rBLH* Brazilian Network of Human Milk Banks, *SBP* Brazilian Society of Pediatrics *CDC* Centers for Disease Control and Prevention, *PPE* Personal Protective Equipment, *RCOG* Royal College of Obstetricians and Gynaecologists, *UENPS* Union of European Neonatal and Perinatal Societies, *UNICEF* United Nations Children’s Fund, *WHO* World Health Organization

Among hygiene measures, washing hands and the use of masks were recommended, but other body surfaces (mother’s face, breasts) that would be in contact with the neonate and may be possibly infected were not specified. Psychological support was available in almost all the hospitals, but one. Hospital discharge within 24 to 48 h was recommended by all but one hospital; one center extended the dyad stay (> 48 h). Prior to the COVID pandemic, the Brazilian recommendations were neonatal discharge > 48 h.

The international guidelines do not detail the home environment recommendations. All hospitals evaluated in this study recommend maintaining breastfeeding and distancing by at least 2 m in the home environment. However, guidance on the management of challenges of the first 15 days after birth, such as neonatal crying and support from the health network were less frequently mentioned in guidelines (Table 3).

**Table 3:** Recommendations in home environment for the management of the mother-infant dyad exposed to SARS-CoV-2

Recommendation	Brazilian hospital guidelines existence (*n* = 24)		Available recommendations in Brazilian and international literature [15–27]
	Yes (%)	No (RA/NR-%)	
Breastfeeding	24 (100)	0 (0)/RA	UENPS, WHO and UNICEF clearly stand in favor; rBLH, SBP, ABM, RCOG and CDC recommend if the mother desires.
Feeding on demand	24 (100)	0 (0)/NR	WHO, RCOG, ABM, SBP, CDC, UNICEF and UENPS do not specify.
Comforting techniques	11 (45.8)	13 (54.1)/NR	WHO, UNICEF, CDC, RCOG, ABM, SBP and UENPS do not specify.
Other person’s neonatal care (not mother’s care)	16 (66.6)	8 (33.3)/NR	CDC, ABM and SPB recommend; RCOG, WHO, UNICEF and UENPS do not specify.
Infant formula	6 (25)	18 (75)/RA	RCOG, ABM, SBP and CDC do not specify; UENPS clearly discourages and recommends expressed fresh mother’s milk; WHO, UNICEF suggests infant formula if breast milk is not possible.
Hygiene measures	24 (100)	0 (0)/RA	WHO, UNICEF, CDC, RCOG, ABM, UENPS, SBP and rBLH allow alcohol gel before handling.
2 m distance	24 (100)	0 (00)/RA	ABM, UENPS, SBP and CDC recommend; RCOG, WHO and UNICEF do not specify.
Separate rooms	4 (16.6)	20 (83.3)/NR	Recommendation not specified for home environment; UENPS, SBP, RCOG, WHO and UNICEF do not specify; ABM recommends as an option; CDC recommends.
Distance between siblings	15 (62.5)	9 (37.5)/NR	WHO, UNICEF, CDC, RCOG, SBP, ABM and UENPS do not specify.
Actions to support breastfeeding	11 (45.8)	13 (54.1)/NR	WHO, UNICEF, UENPS, ABM and SBP do not specify; rBLH recommends suspending educational groups on breastfeeding; RCOG and CDC recommend.
Home visit	4 (16.6)	20 (83.3)/RA	RCOG, ABM, SBP, CDC, WHO and UNICEF do not specify; UENPS discourages.
Telemedicine	11 (45.8)	13 (54.1)/NR	WHO, RCOG, CDC and UENPS encourage; ABM does not specify; rBLH recommends for breastfeeding support and encouragement; UNICEF do not specify.
Reference center to medical evaluation	5 (20.8)	19 (79.1)/NR	UENPS recommends return with 7 days, 14 days and 28 days after discharge; WHO, UNICEF, CDC, RCOG, ABM and SBP do not specify.
Newborn screening in the hospital	11 (45.8)	13 (54.1)/NR	WHO, CDC, RCOG, ABM, SBP, UNICEF and UENPS do not specify.
Psychological call center	9 (37.5)	15 (62.5)/NR	ABM, WHO, UNICEF SBP and UENPS do not specify; CDC and RCOG recommend.

*RA* Recommended against, *NR* There was no description of the recommendation in guidelines, *ABM* Academy of Breastfeeding Medicine, *rBLH* Brazilian Network of Human Milk Banks, *SBP* Brazilian Society of Pediatrics *CDC* Centers for Disease Control and Prevention, *PPE* Personal Protective Equipment, *RCOG* Royal College of Obstetricians and Gynaecologists, *UENPS* Union of European Neonatal and Perinatal Societies, *UNICEF* United Nations Children’s Fund, *WHO* World Health Organization

Home environment support and encouragement for the dyad were variable, telemedicine and newborn screening before discharge were offered in 45.8% of the centers, and home visits were performed only by 16.6% of the centers.

Table 4 shows that hospital breast pumping was allowed (87.5%) as well as fresh mother’s breast milk supply for the neonates (87.5%); however offering the infant expressed breast milk via a feeding line was not recommended (95.8%) and human milk donation was widely forbidden (95.8%). Human milk donation was not recommended to mothers who had not been able to breastfeed because of severe illness. However, the mothers evaluated in our study were in rooming-in with mild symptoms of COVID-19, therefore could perform the technique.

**Table 4:** Recommendations on breastfeeding and human milk handling for the mother-infant dyad exposed to SARS-CoV-2

Recommendation	Brazilian hospital guidelines existence (*n* = 24)		Available recommendations in Brazilian and international literature [15–27]
	Yes (%)	No (RA/NR-%)	
Hospital pumping	21 (87.5)	3 (12.5)/RA	RCOG, WHO, UNICEF, UENPS, SBP, rBLH, ABM and CDC allow.
Bedside pumping	19 (79.1)	5 (20.8)/RA	RCOG, WHO, UNICEF, UENPS, SBP, rBLH, ABM and CDC do not specify.
Fresh mother’s breast milk	21 (87.5)	3 (12.5)	WHO, UNICEF, RCOG, SBP, ABM and CDC suggest as a breastfeeding alternative; UENPS does not recommend if mother is symptomatic; rBLH allows expressing, but for patients in neonatal units the milk must be pasteurized and the milk from mothers in intensive care units must be thrown away.
Feeding line at the breast	2 (8.3)	23 (95.8)/NR	UNICEF, ABM, SBP, CDC UENPS and RCOG do not specify. WHO recommends.
Cup feeding	18 (75)	6 (25)/NR	WHO, UNICEF, ABM, SBP, CDC and UENPS recommend cup or spoon feeding; RCOG does not specify.
Pasteurization	4 (16.6)	20 (83.3)/RA	UENPS discourages, even for preterm babies; SBP and rBLH recommend that for patients in neonatal units the milk must be pasteurized; RCOG, ABM, CDC, WHO and UNICEF do not specify.
Donation permission	2 (8.3)	23 (95.8)/RA	SBP and rBLH don’t allow that these women are donors up to 15 days after the onset of symptoms; ABM, RCOG, WHO, and UNICEF do not specify; CDC forbids.
Home pumping	11 (45.8)	13 (54.1)/RA	WHO, CDC, UNICEF, RCOG, ABM, rBLH and UENPS allow.

*RA* Recommended against, *NR* There was no description of the recommendation in guidelines, *ABM* Academy of Breastfeeding Medicine, *rBLH* Brazilian Network of Human Milk Banks, *SBP* Brazilian Society of Pediatrics *CDC* Centers for Disease Control and Prevention, *PPE* Personal Protective Equipment, *RCOG* Royal College of Obstetricians and Gynaecologists, *UENPS* Union of European Neonatal and Perinatal Societies, *UNICEF* United Nations Children’s Fund, *WHO* World Health Organization

## Discussion

This study evaluated at least one referral hospital in each Brazilian region committed to promoting breastfeeding, and 50% of the services evaluated have a BFHI certification. Regardless of the importance of breastfeeding, the guidelines evaluated do not ensure and protect breastfeeding practices.

The possibility of maternal-fetal virus transmission leading to infection in the neonatal period had been observed in other respiratory diseases before the current pandemic [28]. While the literature on COVID-19 is still limited, the available data has not observed significant infection rates among neonates born from mothers who tested positive for COVID-19; when it occurred, clinical manifestations were generally mild [1–6, 11–14].

As a result of the lack of evidence pertaining to the direct transmission via breast milk, the major concerns about SARS-CoV-2 in addition to evidence of contact and aerosol dissemination with mother and infant in close proximity when breastfeeding, the guideline recommendations are restrictive measures related to breastfeeding which fail to recognise the importance of breastfeeding [15–27].

### Breastfeeding and its socio-economic impact

Undoubtedly, breastfeeding has a positive impact not only on maternal-infant health, but also socio-economically. The WHO highlights the need for public policies for breastfeeding maintenance, as it can prevent 82,300 infant deaths per year worldwide. Nevertheless, even with intense campaigns and publications on the benefits of breastfeeding, only 37% of children are breastfed until 6 months of age worldwide, showing the need for investments in this area [29–31].

The absence of breastfeeding has a negative impact in countries worldwide. In developing societies, which are even more impoverished by the economic crisis triggered by the pandemic, with rising unemployment and falling Gross Domestic Product (GDP); this impact is likely to be greater [32]. Adding to that, total annual global economic losses related to non-breastfeeding are estimated to be between US $ 257 billion and US $ 341 billion, or between 0.37 and 0.70% of the global GDP [8].

Despite the data related to breastfeeding, strategies for protecting this valuable practice during the pandemic have not been adequately developed [15–27]. Although the currently available guidelines discuss prevention of mother-to-child transmission, they do not consider the possibility of weaning the baby and do not foresee actions to minimize the discontinuity of breastfeeding. Countries have prepared themselves to prevent the spread of COVID-19, however the principle of “first do no harm,” whatever the intervention or procedure, the patient’s well-being is the primary consideration, as mentioned by Alison Stuebe in a recent editorial, was not taken seriously [33].

We emphasize that, since COVID-19 is unlike other viral diseases, such as HIV, in which breastfeeding is contraindicated due to the chronic condition of the disease, actions to prevent the transmission of SARS-CoV-2 must be performed within 14 days after birth. However, lack of support during this period combined with maternal anxiety about the disease may cause irreversible consequences on the success of breastfeeding [34, 35].

The vast majority of the hospitals developed their own guidelines (75%) because Brazil is a country with socio-economic differences between the regions, requiring adaptation of the medical literature’s recommendations to their own reality.

### Implications of recommendations

#### Delivery room

By evaluating the recommendations of the protocols for the delivery room, we noticed that most hospitals did not recommend measures to promote breastfeeding for exposed dyads, with a high rate of non-recommendation of skin-to-skin contact (79.1%) and breastfeeding in the first hour after birth (87.5%). These recommendations may lead to dyad separation, with possible impairment of breastfeeding and consequent intense maternal stress, which may contribute to early breastfeeding cessation. They also can contribute to stress to the infant and may hamper an infant’s adaptation to extrauterine life [36]. The concern regarding contamination in the delivery room is related to the active phase of normal delivery, where the mother can disperse droplets. However, pregnant women wear masks according to the guidelines, reducing the spread of particles [37].

We also consider the prohibition of labor companionship as a questionable measure, possibly attributed to prevent crowding in the delivery room. However, the maternal anxiety of being without a partner can be much more harmful, since several publications reinforce the importance of the father or the presence of a companion in the entire process, from birth to breastfeeding [38–41].

There are few guidelines on bathing the neonate, as recommended for other viral conditions. The SARS-CoV-2 may be transmitted through feces, and this measure perhaps should be considered, although bathing is associated with neonatal stress. Only SBP recommends individualizing the bathing after birth [40, 41].

#### Rooming-in

In rooming-in, only one hospital did not allow breastfeeding and separated the dyad. This is not recommended, since separation can compromise support, increase maternal stress, discourage breastfeeding, and increase the consumption of PPE [35, 42].

Hand washing (95.8%) and the use of a surgical mask for breastfeeding (95.8%) were widely recommended in our sample, as well as maintaining a 2-m distance between mother and child (95.8%), possibly making the immediate postpartum period more exhausting, especially when the woman is likely to be in pain.

However, even with restrictions that require more effort from the puerperal woman, most services did not allow a room companion room (95.8%), or a family member to be a facilitator of breastfeeding. Responsive feeding, changing diapers, and bathing demand constant hygiene from the puerperal women and being alone would probably make the correct execution of the protection measures more unlikely and, again, contribute to maternal tiredness and anxiety. Mental health during the pandemic is already a concern of health agencies, and services must plan measures to prevent worsening of mental health [41].

Additionally, the permission of a companion for the dyad could facilitate the health education of a member of the family support network, who would be adequately trained to provide help during the period of isolation at home, avoiding contamination and encouraging breastfeeding, since the support is fundamental to its success [41].

Shared decision-making about breastfeeding with the family was adopted in 75% of the hospitals, however the protocols were not individualized. When adopting the same recommendation for all mothers, they did not consider the role of breastfeeding in that family and did not assess the dyad’s socio-economic situation.

The dyad’s assessment considering a bio-psycho-social approach would possibly avoid the implementation of useless measures in a hospital environment, since these recommendations cannot be followed in the home environment, especially in developing countries where several people inhabit places with insufficient hygiene conditions [31, 43].

Another interesting aspect is related to surface cleaning. Mothers with COVID-19, due to the common symptoms—coughing, sneezing, and scratching their faces—can increase the chance of having their face, clothes, and breasts potentially contaminated. However, there are no specific recommendations for cleaning these surfaces.

Breastfeeding support and guidance on breastfeeding with COVID-19 were offered in most of the services evaluated; however, in 79.1% of Brazilian hospitals, discharge took place between 24 and 48 h, a period when breastfeeding is often not yet well established, mainly in primiparous mothers or those with breastfeeding-related anxieties. Additionally, guidance for mothers and family members were more specific to prevent infection than to promote and support breastfeeding [15–27, 30, 44, 45].

### Home environment

Regarding the home environment, the guidelines are standardized: all of them allow breastfeeding with hygiene measures. However, the recommendations are vague on how to proceed when the newborn is crying, with only 45.8% of the evaluated guidelines guiding how the mother should manage the crying of the newborn during the isolation period. The guidance on how to console the neonate should be seriously considered since crying is referred to as one of the leading causes of early cessation of breastfeeding, and adequate management can alleviate maternal-fetal stress [30–35].

The prescription of infant formula and guidance for neonatal care to be executed by other people was performed in approximately 20% of the evaluated hospitals; measures that can lead to weaning, and the lack of bond between child and mother. Concerning siblings, 62.5% of services recommended that children be separated from the dyad, a situation that probably would not be possible in socially vulnerable families, and possibly contribute to maternal anxiety [30, 35, 41, 42].

Another significant difficulty observed is the support from the primary care health service. Home visits were conducted by only 16.6% of the services; telemedicine was used by 45.8% and psychological care by telemedicine was only used by 37.5%. The lack of recommendations for adequate home support is worrying, since this type of measure would be fundamental for the promotion of breastfeeding [46].

Despite the recommendations to avoid follow-up in primary care,79% of participating hospitals recommend review at the primary care unit if there is a problem with the dyad rather than return to the referral health service.

In an attempt to contribute to the dyad isolation, 45.8% of hospitals performed the neonatal screening in a hospital environment. Probably, when this possibility is not offered, there will be possible delays in the diagnosis of neonatal diseases.

### Breast pumping and human milk donation

Regarding the handling and donation of human milk, we noticed guidance for breast pumping in a hospital environment (87.5%), although these mothers cannot be milk donors during the isolation period, probably due to the concerns of contamination [47].

Most hospitals recommended that fresh mother’s breast milk must be offered to the newborn (87.5%), preferably by cup (75%). Only 8.3% of hospitals allowed use of a feeding line possibly not providing the stimulus to lactation through neonatal suction during feeding [44].

Concerning limitations of the study, we evaluated only the most important Brazilian hospitals; therefore, it perhaps does not represent the Brazilian reality in its entirety. We also only analyzed guidelines during the COVID-19 outbreak and cannot be sure of actual practices in these participating hospitals.

Lack of breastfeeding knowledge is significant, and so are the concerns regarding the pandemic’s impact on breastfeeding. Among the international protocols evaluated (WHO, CDC, UNICEF, RCOG, UENPS, and ABM) and national agencies (SBP and rBLH), we observed that the recommendations with the highest agreement between them are those related to preventing infection by SARS-COV-2 of the newborn. Nevertheless, there is no guidance on supporting breastfeeding in pandemic situations or how to continue implementing the actions recommended by breastfeeding protection policies [15–27].

Therefore, to investigate the management of breastfeeding in the dyads exposed to SARS-CoV-2, we prepared a prospective multicenter study, with the 24 Brazilian hospitals evaluated in this study (BRACOVID Project). The purposes are to assess the impact of the COVID-19 guidelines on breastfeeding rates, evaluate maternal mental health, analyze the feasibility of implementing the proposed hygiene measures in the home environment, and the clinical evolution of breastfed neonate born of mothers with COVID-19. A part of this study is still being carried out; final data collection was planned to be completed by September 2020.

The disagreement between international guidelines and the impact of this fact has been discussed in the scientific community [48–50]. The absence of recommendations on breastfeeding support during the pandemic led to difficulties in developing standards among hospitals in different regions of Brazil and other countries worldwide. The lack of a global discussion on supporting and protecting breastfeeding during the pandemic can generate incalculable damage to maternal and child health, leading to psychological, social, and economic costs, especially in the most vulnerable populations.

## Conclusions

We conclude that the evaluated guidelines are not able to support the required actions to encourage breastfeeding, as recommended by well-known protecting policies. Regarding COVID-19 and breastfeeding, an important question remains—what are the repercussions for the lack of support and promotion of breastfeeding in the COVID-19 era? It is reasonable to consider that if protective measures and incentives for breastfeeding are not taken into consideration during this time, the effects of the pandemic will reflect on children’s health for decades.

The dyad exposed to SARS-CoV-2 have an increased risk of early cessation of breastfeeding and a situation of extreme anxiety. Therefore, we understand that our role as neonatologists and other health professionals involved in this process is to encourage breastfeeding, to recognize the risk of early cessation of breastfeeding, and to plan practical support actions.

It is essential that the scientific community discuss and look for effective ways to promote breastfeeding, not only by allowing it but also looking for ways to guarantee its practice. Protocols for the containment of COVID-19 must be realistic and adequate, designed to prevent neonatal infection and with special attention in promoting and supporting breastfeeding during the pandemic.

## Data Availability

The datasets used and analyzed during the current study are available from the corresponding author on reasonable request.

## Abbreviations

ABM: Academy of Breastfeeding Medicine
CDC: Centers for Disease Control and Prevention
PPE: Personal Protective Equipment
RCOG: Royal College of Obstetricians and Gynaecologists
rBLH: Brazilian Network of Human Milk Banks
SBP: Brazilian Society of Pediatrics
SIN: Society of Italian Neonatology
UENPS: Union of European Neonatal and Perinatal Societies
UNICEF: United Nations Children’s Fund
WHO: World Health Organization
